# Dinutuximab beta effectively treats Ewing sarcoma when combined with chemotherapy

**DOI:** 10.1016/j.isci.2025.114449

**Published:** 2025-12-16

**Authors:** Roberta Frapolli, Marina Meroni, Ezia Bello, Lorenza Pirona, Elisa Callegari, Valentina Kebede, Isabella Pellerani, Simone Canesi, Eugenio Scanziani, Uta Dirksen, Patrizia Angelico, Stefano Biondi, Matteo Malinverno

**Affiliations:** 1 Laboratory of Cancer Pharmacology, Department of Experimental Oncology, Instituto di Ricerche Farmacologiche Mario Negri IRCCS, Milan, Italy; 2 Preclinical Group, R&D, Recordati S.p.A., Milan, Italy; 3 Mouse & Animal Pathology Laboratory (MAPLab), UniMi Foundation, Milan, Italy; 4 Department of Veterinary Medicine and Animal Sciences, University of Milan, Lodi, Italy; 5 Pediatrics III, University Hospital of Essen, West German Cancer Center, National Center for Tumor diseases (NCT-West), German Cancer Consortium (DKTK), 45122 Essen, Germany

**Keywords:** health sciences, pharmaceutical science

## Abstract

Ewing sarcoma is a rare and aggressive cancer of the bone and soft tissues primarily affecting children and young adults. Prognosis for patients with metastatic or recurrent disease remains poor despite intensive multimodal therapy, highlighting the need of novel therapeutic approaches. The disialoganglioside GD2 is highly expressed on Ewing sarcoma cells, making this tumor eligible for anti-GD2 immunotherapy with dinutuximab beta. Through *in vitro* and *in vivo* approaches, this study demonstrated that dinutuximab beta effectively suppressed tumor growth by 60% (*p* = 0.0135) and improved survival rate by 68% (*p* = 0.0006) in a mouse model xenograft. The combination therapy with doxorubicin demonstrated superior efficacy compared to monotherapy, with enhanced tumor suppression (86%; *p* = 0.0009) and an extension of survival rate (146%; *p* = 0.000025). This study showed that dinutuximab beta, particularly in combination with standard chemotherapy, offers a promising approach to improve outcomes for high-risk Ewing sarcoma patients, providing a more effective alternative to current treatments.

## Highlights

• Dinutuximab beta inhibits tumor growth and prolongs survival in a murine Ewing sarcoma model
• Combining dinutuximab beta with standard chemotherapy elicits synergistic anti-tumor effect
• Dinutuximab beta acts by recruiting immune effector cells that induce tumor cell death
• Dinutuximab beta with chemotherapy may improve outcomes and survival in Ewing sarcoma patients

## Graphical abstract

**Figure:**
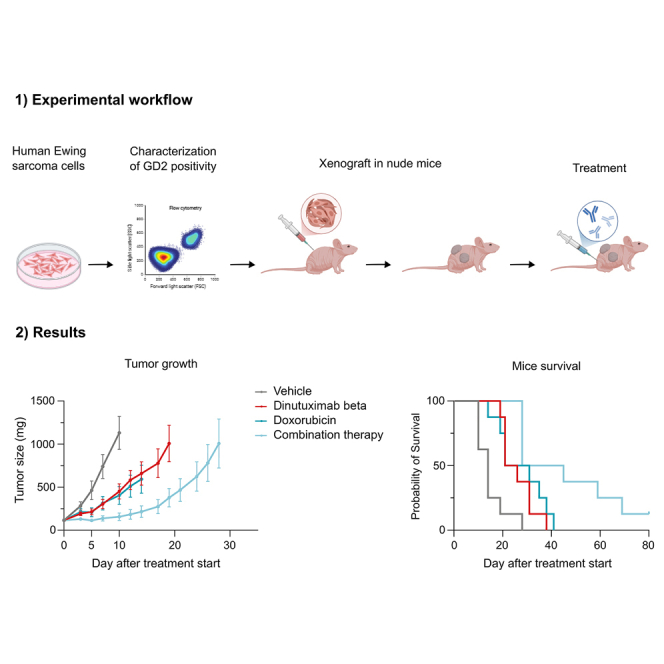

## Introduction

Ewing sarcoma (EwS) is a rare and aggressive form of cancer that affects the bones and soft tissues, and it is estimated that around 250 people in the United States and 600 in Europe are diagnosed every year. EwS mostly affects children and AYAs (adolescent and young adults) with a peak incidence at the age of 15 years, and is slightly more common in male than female patients (sex ratio of 3:2). EwS occurs more often in European descendant populations with an estimated incidence of ∼1.5 cases per million children and AYAs. The estimated incidence in Asian and African populations is much lower, with annual rates of ∼0.8 and ∼0.2 cases per million children, respectively.^1^

EwS can arise in any bone of the body, but most commonly in the pelvis, femur, tibia, humerus, and thoracic wall. EwS can also occur in the soft tissues, such as the muscles, nerves, fat, or blood vessels, in which case it is called extraosseous EwS.

At the genetic level, the hallmark of EwS is a chromosomal rearrangement that causes the EWS gene to join with one of the various ETS transcription factors, with FLI1 being the most frequent partner. The fusion protein acts as an aberrant transcription factor that drives the expression of oncogenic genes and suppresses the expression of tumor suppressor genes.^2^^,^^3^ The current standard of care for patients with EwS consists of multimodal therapy, including multiagent chemotherapy and local treatment modalities as surgery, radiotherapy, and chemotherapy. However, despite the intensive treatment, the prognosis of patients with EwS remains poor, especially for those with metastatic or recurrent disease. The 5-year survival rate for patients with localized disease is around 70%, while for those with metastatic disease it drops to 20%–30%. Moreover, the chemotherapy regimen is associated with significant toxicity and long-term sequelae, such as cardiotoxicity, nephrotoxicity, infertility, and secondary malignancies.^1^^,^^3^ Therefore, there is an urgent need for new therapeutic strategies that can improve the outcome and the quality of life of patients with EwS, by increasing the efficacy and reducing the toxicity of the treatment. One promising approach is the combination of chemotherapy with immunotherapy, which aims to harness the immune system to recognize and eliminate the tumor cells. To be effective, immunotherapy requires the presence of antigens that are predominantly expressed in the tumor cells. One of these antigens is disialoganglioside GD2 which is highly express by almost all the tumors of neuroectodermal origin, such as neuroblastoma, osteosarcoma, melanoma, and small cell lung cancer. The GD2 antigen is not expressed by normal tissues, except for the peripheral nerves and the brain. This makes it an attractive target for immunotherapy, as it can distinguish the tumor cells from the normal cells.^4^

EwS has been studied for GD2 expression, and the results ranged from no detectable surface expression to diffused and/or intense staining in some tumors.^5^ More specifically, GD2 expression levels ranged from 40% to 90% from diagnostic biopsy samples of EwS.^6^ Kailayangiri et at., detected GD2 expression by immunofluorescence staining in ten of 19 EwS cell lines and three of three primary cell cultures, concluding that surface expression of GD2 is characteristic of EwS and that GD2 provides an appropriate target antigen for therapeutic strategies to eradicate micro metastases and reduce the risk of recurrence in patients with high-risk disease^7^^,^^8^

Dinutuximab beta is a monoclonal antibody that targets the GD2 antigen expressed by the tumor cells. Dinutuximab beta binds to the GD2 antigen and recruits the immune effector cells, such as natural killer (NK) cells and macrophages, to mediate antibody-dependent cellular cytotoxicity (ADCC) and complement-dependent cytotoxicity against the tumor cells.

Dinutuximab beta is indicated for high-risk neuroblastoma in patients over 12 months old, who have responded at least partially to prior treatments, or who have had neuroblastoma relapsed or refractory, with or without any remaining disease.

According to an anecdotal report, three patients who had newly diagnosed, metastatic, GD2-positive EwS received dinutuximab beta along with the standard regimen of consolidation chemotherapy. All patients tolerated the treatment well and reached complete remission, with no signs of recurrence, thus indicating dinutuximab beta as a promising immunotherapy for EwS.^9^ However, the efficacy of dinutuximab beta in treating EwS and the underlying molecular and cellular mechanisms of its therapeutic action have not been previously investigated. Utilizing both *in vitro* and *in vivo* approaches, we provide evidence that dinutuximab beta effectively suppresses EwS growth, functioning both as a monotherapy and in combination with the standard chemotherapeutic agent doxorubicin.

## Results

### Dinutuximab beta induces ADCC in GD2-positive EwS cells *in vitro*

**Figure 1:**
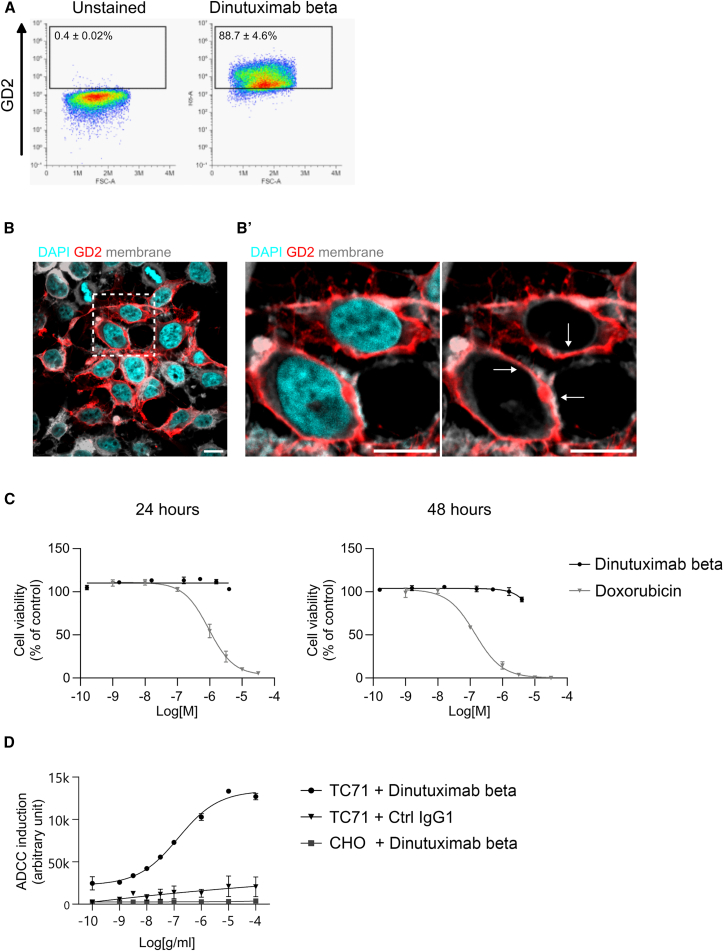
Dinutuximab beta induces ADCC in GD2-positive EwS cells *in vitro* (A) FACS analysis on TC-71 either stained or not with dinutuximab beta conjugated to IRDye800CW fluorophore. (B) Confocal images of TC-71 cells stained, without permeabilization, for GD2 and plasma membrane. Nuclei were counterstained with DAPI. (B′) Magnification of the cropped area. Arrows indicate GD2 expressed on the plasma membrane. Scale bars represent 10 μm. (C) Cell viability assay. TC-71 cells were treated with either dinutuximab beta or doxorubicin at the indicated concentrations for 24 or 48 h. Cell viability is shown as % of viable cells relative to the control group (vehicle treated). (D) Antibody-dependent cellular cytotoxicity was evaluated with the ADCC reporter bioassay. TC-71 cells were treated with either dinutuximab beta or IgG isotype, at the indicated concentrations. As negative control, GD2-negative CHO-K1 cells were treated with dinutuximab beta at the same concentrations. Data are expressed as arbitrary value of signal intensity.

The anti-tumor activity of dinutuximab beta was investigated *in vitro* using the human EwS cell line TC-71. These cells harbor the EWS/FLI1 type 1 translocation and exhibit GD2 expression levels broadly representative of other GD2-positive EwS lines, which typically show intermediate GD2 expression compared to neuroblastoma cells, known for their very high GD2 levels.^7^^,^^8^ FACS analysis performed with dinutuximab beta showed that 88.65 ± 4.55% of TC-71 cells were GD2-positive, confirming both the GD2 expression of the cell line and the ability of the therapeutic antibody to bind them (Figure 1A). We also confirmed that TC-71 cells express GD2 at significantly lower levels compared to neuroblastoma cells (Figure S1). Finally, surface expression of GD2 was confirmed by confocal imaging (Figure 1B). The effect of dinutuximab beta on TC-71 cells was then investigated in a cell viability assay. The treatment with dinutuximab beta for up to 48 h did not induce reduction of cell viability, even at the highest dose of 4.3 μM (Figure 1C). These cells showed sensitivity to doxorubicin, with an EC50 of 0.92 μM and 0.14 μM at 24- and 48-h post-treatment, respectively (Figure 1C). Conversely, dinutuximab beta was able to activate effector cells in an antibody-dependent-cellular cytotoxicity *in vitro* assay (Figure 1D). Taken together, these data show that dinutuximab beta does not affect directly the viability and proliferation rate of EwS cells, but it induces strong cytotoxicity by recruitment and activation of the immune system, in particular of the effector cells such as NK cells.^10^^,^^11^

### Dinutuximab beta inhibits tumor growth and increases survival *in vivo*

**Figure 2:**
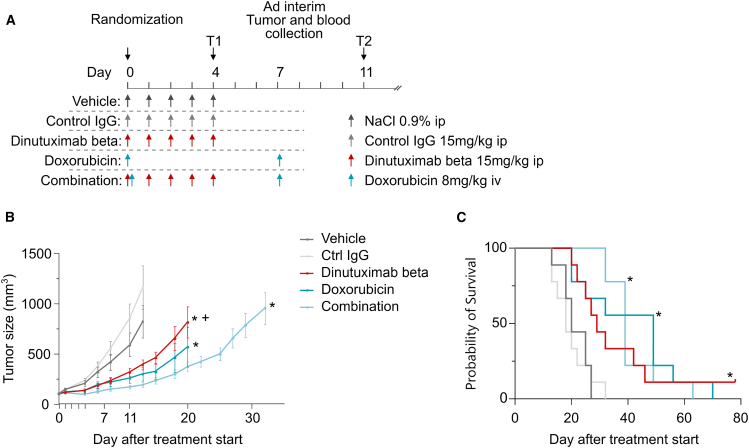
Dinutuximab beta inhibits tumor growth and increase survival *in vivo* (A) Diagram of the *in vivo* experiment. The diagram indicates the dosage and schedule of the various drugs administered to the animals as well as the two time points at which the tumors and blood samples from the satellite groups were collected. (B) Tumor growth curves obtained in TC-71 tumor bearing mice treated with dinutuximab beta, doxorubicin, and their combination. Tumor size is expressed as mg. Mixed-effects model was applied; ∗*p* < 0.05 vs. vehicle; + *p* < 0.01 vs. combination. Complete statistical analysis is reported in Table S1. (C) Survival curves obtained in TC-71 tumor bearing mice treated with dinutuximab beta, doxorubicin and their combination. Restricted mean survival time analysis was applied; ∗*p* < 0.01 vs. vehicle. Complete statistical analysis is reported in Table S2.

The anti-tumor activity of dinutuximab beta was tested on a xenograft murine model of EwS established by injecting subcutaneously TC-71 cells into nude athymic mice, as previously described.^12^^,^^13^ Nine days after tumor inoculation, mice were randomized into the following five treatment groups: vehicle, control IgG, dinutuximab beta, doxorubicin, and combination, as described in Figure 2A. Animals in the dinutuximab beta group received daily intraperitoneal (i.p.) injections of dinutuximab beta at 15 mg/kg for five consecutive days, from day 0 to day 4 post randomization; animals in the doxorubicin group received the drug intravenous (i.v.) at 8 mg/kg on day 0 and day 7, while in the combination group, the animals received the two drugs following the same scheme as the single treatments. Two control groups were included: the first group received five consecutive daily i.p. injections of the vehicle, while the second control group received five consecutive daily i.p. injections of the control IgG isotype at 15 mg/kg. In parallel, satellite groups for each treatment were sacrificed on day 4, 4 h after the last dose of dinutuximab beta, and on day 11, 96 h after the second dose of doxorubicin, for pharmacodynamic and pharmacokinetic studies.

Tumor growth curves obtained during the experiment are reported in Figure 2B. Compared with the vehicle group, dinutuximab beta as single agent significantly inhibited tumor growth (*p* = 0.0343 vs. vehicle), but this effect was limited to the treatment period, then tumor regrowth occurred. The best mean tumor volume ratio between the treated and control groups (T/C) was 48% on day 13. The treatment with doxorubicin had a slightly better efficacy with a T/C of 36% on day 13 (*p* = 0.0215 vs. vehicle). The combination of dinutuximab beta and doxorubicin improved the efficacy of the antibody alone, with a best T/C of 23% on day 13. This improvement is statistically significant when compared with dinutuximab beta administered alone (*p* = 0.0067). Complete statistical analysis of the tumor growth curves is reported in Table S1.

The analysis of the survival curves (Figure 2C) substantially confirmed the treatment efficacy, with increment of life span (ILS) of 45%, and one out of nine mice being tumor-free at the end of the observation period, in the group of mice treated with dinutuximab beta alone. The combined treatment further improved mice survival, resulting in an ILS of 95%. In the doxorubicin group, an ILS of 145% was recorded, probably related to the variability of the tumor regrowth after treatment. Complete statistical analysis of the tumor growth curves is reported in Table S2.

**Figure 3:**
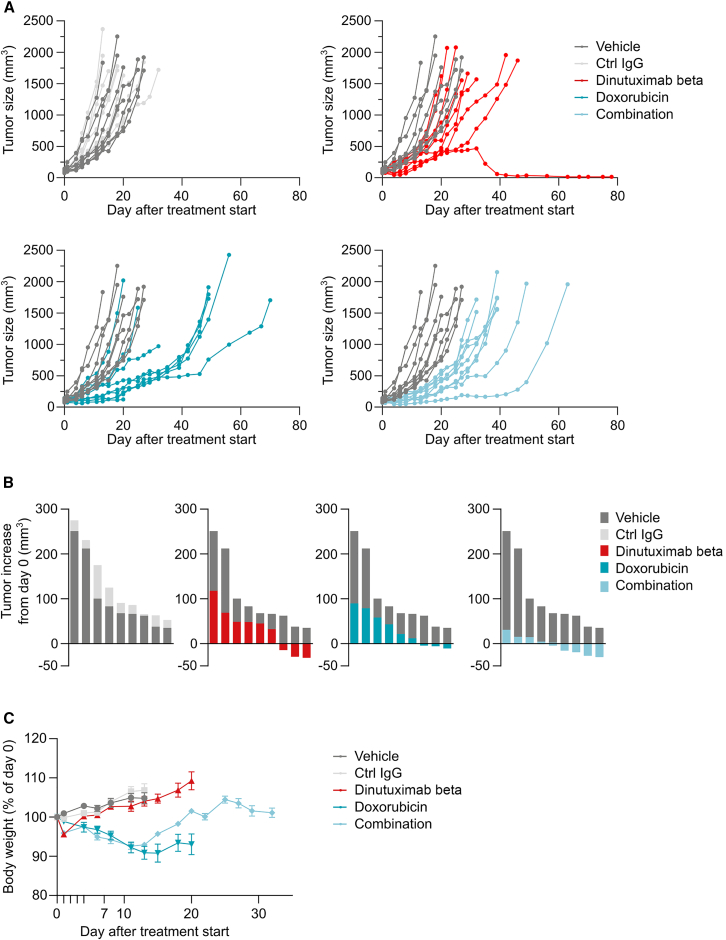
Dinutuximab beta induces partial tumor shrinkage (A) Growth curves of individual tumors. Tumor size is expressed as mm^3^. (B) Change in tumor size for each tumor at day 4 compared to day 0. Each bar represents the size of one tumor. Data are shown as the difference between tumor size at day 4 (in mm^3^) and tumor size at day 0 (in mm^3^). In the dinutuximab beta, doxorubicin and combination group, three out of nine, three out of nine, and five out of nine tumors decreased in size, respectively. (C) Plot showing average body weight of TC-71 tumor bearing mice treated with dinutuximab beta, doxorubicin and their combination. Data are shown in grams.

Figure 3 shows the growth curve of each individual tumor over time. All the tumors in the vehicle and control IgG groups increased in size over the treatment period. Conversely, the treatments caused varying degrees of partial tumor shrinkage. Specifically, on day 4, the last day of dinutuximab beta treatment, both dinutuximab beta and doxorubicin as single treatments caused shrinkage of three out of nine (30%) tumors, while the combined treatment caused shrinkage of five out nine tumors (55%) (Figure 3B). Subsequently, tumors in the doxorubicin and combination groups re-grew slower than those in the dinutuximab beta group, likely due to the second dose of doxorubicin administered on day 7.

The treatment with dinutuximab beta was well tolerated, with no body-weight loss (BWL) or other drug-related clinical signs of distress (Figure 3C). On the other hand, doxorubicin, both as monotherapy and in combination, caused significant BWL averaging around 10%, which is considered a sign of moderate suffering. In the doxorubicin group, one mouse was found dead for unknown reasons after a BWL of about 10%.

### Dinutuximab beta promotes immune effector cell recruitment and induces tumor cell death

**Figure 4:**
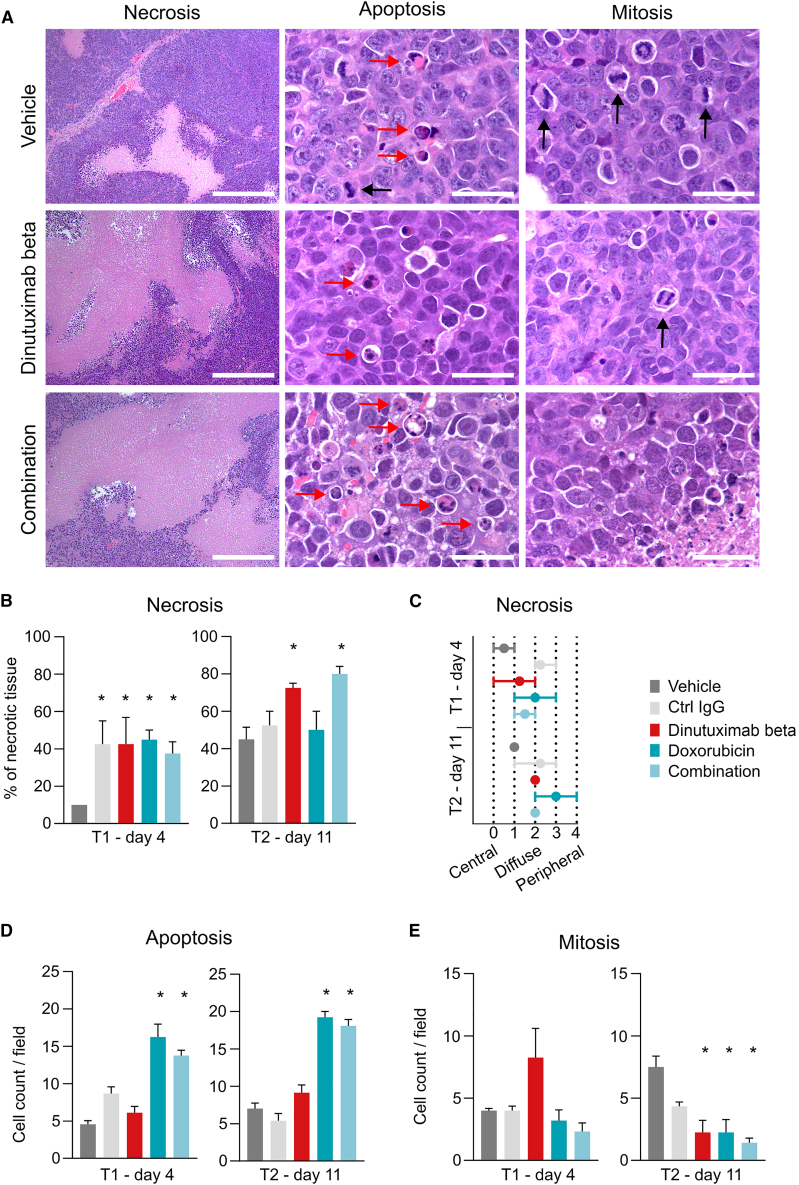
Dinutuximab beta inhibits tumor cell proliferation and induces necrosis (A) Representative pictures of tumors stained by hematoxylin and eosin (H&E), collected on day 11 (T2) after treatment start. Necrosis: scale bars represent 50 mm; limited areas of coagulative necrosis surrounded by viable tumoral tissue are visible in the control, while dinutuximab beta and combination treated samples display large areas of coagulative necrosis in the presence of limited islands of viable tumoral tissue. Apoptosis and mitosis: scale bars represent 5 mm; red arrows indicate apoptotic tumoral cells characterized by shrunken cytoplasm and dark nuclear round bodies, while black arrows indicate mitotic tumoral cells. (B) Evaluation of necrotic tissue expressed as % of necrotic tissues compared to total tumor tissue. Data are presented as mean ± S.E.M. T1 and T2, *p* < 0.05 among groups (Kruskal-Wallis test), ∗*p* < 0.05 vs. vehicle (uncorrected Dunn’s test). (C) Graphical representation of the predominant localization of necrosis within the tumor. Data are presented as mean ± S.E.M. A score was assigned depending on the primary distribution pattern of necrosis. (See STAR Methods section Table S3 for a description of the scoring system). (D) Count of apoptotic cells per field. Data are presented as mean ± S.E.M. T1 and T2, *p* < 0.01 among groups (Kruskal-Wallis test), ∗*p* < 0.05 vs. vehicle (uncorrected Dunn’s test). (E) Count of mitotic cells per field. Data are presented as mean ± S.E.M. T1, *p* = 0.122 among groups (Kruskal-Wallis test); T2, *p* < 0.05 among groups (Kruskal-Wallis test), ∗*p* < 0.05 vs. vehicle (uncorrected Dunn’s test).

Tumors of the satellite groups collected at the two time points (see Figure 2A) underwent histopathological evaluation after hematoxylin & eosin staining (Figure 4A). All samples showed signs of necrosis, with more necrotic tissue in control animals at T2 than in control animals at T1, as expected due to massive tumor growth. The extension of necrotic areas was greater in all groups of treatment in comparison with controls at both timepoints. This increase was particularly evident in dinutuximab beta and combination groups at T2 (Figure 4B). Moreover, in untreated tumors, necrosis was primarily localized to the central region of the specimens at both time points, consistent with expected patterns of spontaneous necrosis. In contrast, tumors that received treatment, particularly in the dinutuximab beta group, exhibited a diffuse distribution of necrosis throughout the entire tumor mass (Figures 4C and S2, Table S3). This finding confirms that the treatment with dinutuximab beta effectively reaches the entire tumor mass.

The mitotic and apoptotic cell count revealed significant differences across the various treatment groups. Notably, there was an increase in the mitosis count in the group treated with dinutuximab beta at T1, although statistical significance was not reached (*p* = 0.122, Kruskal-Wallis test). While this observation may seem contradictory to the overall tumor reduction, it can be explained by the possibility of tumor cells being arrested in the S and G2/M phases, thus not actively proliferating but appearing morphologically similar to mitotic cells. By T2, there was a marked decrease in the mitotic cell count across all treatment groups, which correlates well with a strong reduction in tumor growth (Figure 4D). Apoptosis was increased in the group that received doxorubicin, either as monotherapy or in combination, at both timepoints (Figure 4E), as expected due to its mechanism of action.^14^ Taken together these data suggest that both doxorubicin and dinutuximab beta (and their combination) inhibit tumor cell proliferation and induce cell death.

**Figure 5:**
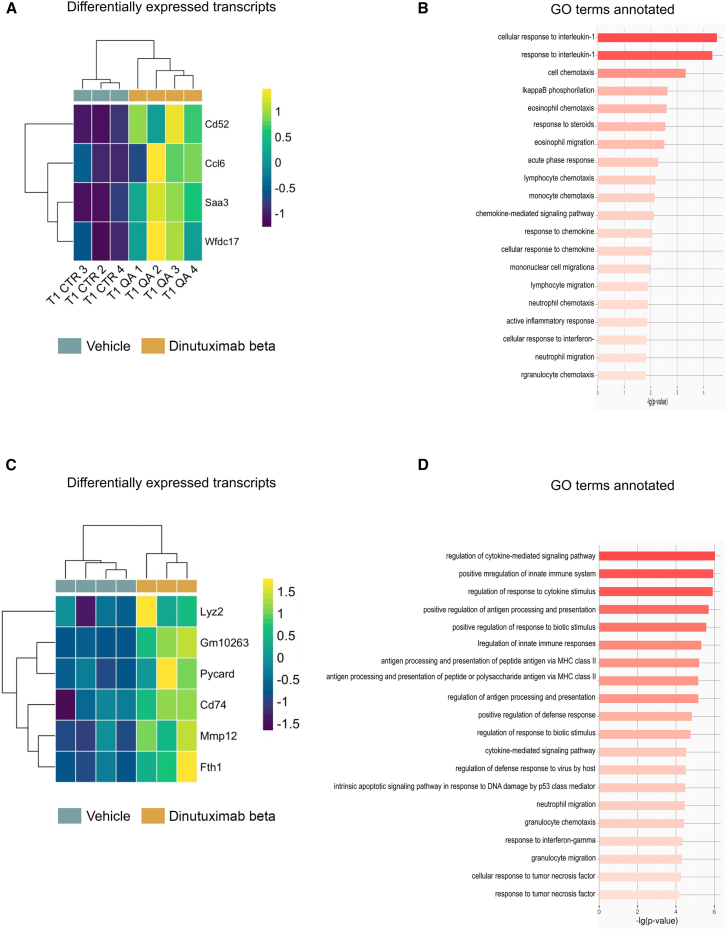
RNAseq analysis revealed infiltration of immune effector cells into dinutuximab beta-treated tumors Differential expression and gene ontology analysis of murine transcripts (A) Heatmap showing the differentially expressed transcripts between vehicle and dinutuximab beta treated groups at T1. Transcripts with adjusted *p* value <0.05 are shown. (B) Gene ontology (GO) analysis of the protein-coding mRNA differentially expressed between vehicle and dinutuximab beta-treated groups at T1. The bar plot shows the top 20 significantly enriched biological process GO terms (*p* value <0.05). (C) Heatmap showing the differentially expressed transcripts between vehicle and dinutuximab beta treated groups at T2. Transcripts with adjusted *p* value <0.05 are shown. (D) Gene ontology analysis of the protein-coding mRNA differentially expressed between vehicle and dinutuximab beta treated groups at T2. The bar plot shows the top 20 significantly enriched biological process GO terms (*p* value <0.05).

To gain a more comprehensive understanding of the mechanism of action of dinutuximab beta, we performed RNA sequencing analysis on tumor biopsies from satellite groups treated with either vehicle or dinutuximab beta, at both time points. We leveraged the difference in species origin to distinguish transcripts derived from the tumor (human) from those originating from host-infiltrating cells (murine). As expected, the majority of transcripts, ranging from 85% to 95%, were of human origin, while only 1.5%–5.8% derived from the host (Figure S3). The analysis of the murine transcriptome identified four differently expressed transcripts (adjusted *p* value <0.05) at T1 (Figures 5A, S4A and S4B), all upregulated following treatment with dinutuximab beta. Gene ontology analyses (Figure 5B) revealed enrichment for biological processes involved in the response to cytokines responsible for triggering the acute phase of inflammation, as well as the immune response, including interleukin-1 (IL-1) and interferon-gamma. Parallelly, these genes are positively associated to chemotaxis and migration of immune cell populations, including lymphocytes, monocytes and granulocytes. These data confirm that dinutuximab beta induces innate immune response through secretion of cytokines and recruitment of effector cells.

**Figure 6:**
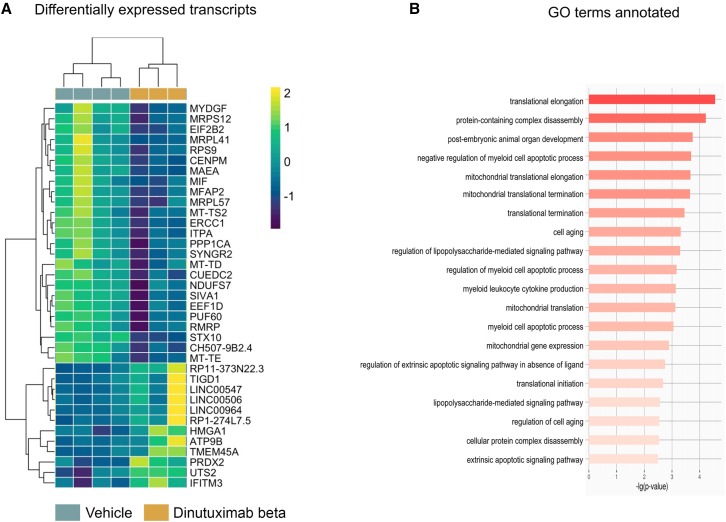
RNAseq analysis revealed biological processes involved in cell cycle arrests and cell death in tumors treated with dinutuximab beta Differential expression and gene ontology analysis of human transcripts (A) Heatmap showing the differentially expressed transcripts between vehicle and dinutuximab beta treated groups at T2. Transcripts with adjusted *p* value <0.05 are shown. (B) Gene ontology analysis of the protein-coding mRNA differentially expressed between vehicle and dinutuximab beta-treated groups at T2. The bar plot shows the top 20 significantly enriched biological process GO terms (*p* value <0.05).

At T2, five protein-coding mRNA and one lncRNA up-regulated in the dinutuximab beta group were identified (Figures 5C, S4C, and S5D). Here also, the gene ontology revealed enrichment for biological processes involved in response to cytokines and activation of the immune response, particularly of the adaptive immune system, as revealed by genes involved in antigen processing and presentation via MHC class II (Figure 5D). The analysis on human transcriptome identified two transcripts differentially expressed at T1 (Figures S5A and S5B). HERC2P8 is pseudogene with no know biological function, while IFITM3 is a gene that has been shown to modulate the tumor microenvironment by promoting T cell infiltration, which is essential for antitumor immunity.^15^ In contrast, its deficiency disrupts antigen processing and presentation.^16^ Recently, IFITM3 has also been identified as a potent biomarker for predicting immunotherapy efficacy in some cancers and its overexpression has been shown to enhance antitumor immunity.^17^ At T2 37 (12 upregulated and twenty-five downregulated following treatment with dinutuximab beta) differentially expressed transcripts were identified (Figures 6A, S5C, and S5D). Of these, twenty-nine transcripts were protein-coding mRNA, showing enrichment in biological processes such as translational elongation and termination, cell aging and apoptosis (Figure 6B). Moreover, IFITM3 was also upregulated in dinutuximab beta-treated tumors at T2. Interestingly, this upregulation is paralleled by enrichment of gene involved in antigen processing and presentation by murine effector cells infiltrating the tumor, suggesting a potential key role of this gene in the response to dinutuximab beta. These findings reinforce the hypothesis that the antitumor effect of dinutuximab beta is mediated through the arrest of the cell cycle and the induction of cell death.

### A second cycle of treatment enhances the efficacy of combination therapy

As previously shown in Figure 2, dinutuximab beta as a single agent induced a significant inhibition of tumor growth, which was, however, limited to the duration of the treatment. Subsequently, the tumor began to grow again. Conversely, the treatment with doxorubicin had a slightly longer effect on tumor growth, which can be attributed to the second dose given on day 7. In fact, the growth curves of tumors treated with dinutuximab beta or doxorubicin begin to diverge after day 8.

**Figure 7:**
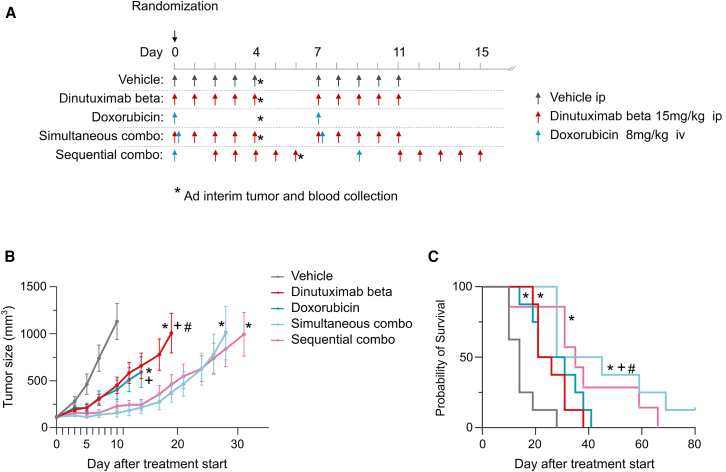
A two-cycle regimen enhances the effectiveness of combination therapy (A) Diagram of the *in vivo* experiment. The diagram illustrates the dosage and treatment scheduled of the various drugs administered to the animals. The asterisks indicate the time points at which the tumors and blood samples from the satellite groups were collected. (B) Tumor growth curves obtained in TC-71 tumor bearing mice treated with dinutuximab beta, doxorubicin and their combination. Tumor size is expressed as mg. Mixed-effects model was applied; ∗*p* < 0.05 vs. vehicle; + *p* < 0.05 vs. simultaneous combination; #*p* < 0.05 vs. sequential combination. Complete statistical analysis is reported in Table S4. (C) Survival curves obtained in TC-71 tumor bearing mice treated with dinutuximab beta, doxorubicin and their combination. Restricted mean survival time analysis was applied; ∗*p* < 0.01 vs. vehicle; + *p* < 0.05 vs. doxorubicin; #*p* < 0.05 vs. dinutuximab beta. Complete statistical analysis is reported in Table S5.

As commonly observed with monoclonal antibodies, dinutuximab beta has a long half-life of 59 h (4.5 days) in nude athymic mice.^18^ The concentration of dinutuximab beta was measured in the plasma of tumor-bearing mice from the satellite groups, collected on day 4 and day 11 after treatment start. It rapidly decreased from 933.1 ± 280.2 μg/ml 4 h after the last administration (on day 4) to 115.2 ± 36.2 μg/ml on day 11, with a reduction of 87.7% in 7 days (T_1/2_ 3.9 days). This rapid decrease of tumor exposition to dinutuximab beta can explain the drug’s short-term efficacy on tumor growth. To address this limitation, we designed a follow-up experiment (Figure 7A) in which the treatment with dinutuximab beta was repeated a second time, 7 days after the start of the first cycle (day 7–11). Similarly, the combination therapy was administered in two cycles. Moreover, with the aim of enhancing the synergistic efficacy of combination therapy, we included a group of animals that received a different treatment regimen. In this group, the animals were initially treated with doxorubicin on day 0. Following a two-day interval, a 5-day cycle of dinutuximab beta treatment started, spanning from day 2 to day 6. This regimen was then repeated for a second cycle, extending from day 9 to day 15. From now on, the two combination regimens will be referred to as “simultaneous combination” and “sequential combination”, respectively. The detailed experimental scheme is shown in Figure 7A. The tumor growth curves (Figure 7B) confirm the efficacy of dinutuximab beta as single agent, and indicate that, when administered in two cycles, it is as effective as doxorubicin in inhibiting tumor growth. In fact, the two curves are superimposable, with the best T/C being 40% (*p* = 0.0135 vs. vehicle) and 36% (*p* = 0.0189 vs. vehicle; *p* = 0.7417 vs. dinutuximab beta) on day 10 for the dinutuximab beta and doxorubicin group, respectively. The effect of dinutuximab beta was more evident during the first cycle of treatment, then regrowth occurred, and the second cycle had only a minimal effect in delaying tumor growth.

Consistently with what observed in the first experiment, the simultaneous combination of dinutuximab beta and doxorubicin administered in two cycles improved the efficacy of the antibody alone, with the best T/C, on day 10, of 14% (*p* = 0.0009 vs. vehicle). This improvement was statistically significant compared to the use of dinutuximab beta (*p* = 0.0106) and doxorubicin (*p* = 0.0481) as monotherapies. The two distinct combination regimens showed comparable efficacy, with the best T/C being 18% (*p* = 0.0018 vs. vehicle, p0.832 = vs. simultaneous combination) for the sequential combination group. Complete statistical analysis of the tumor growth curves is reported in Table S4.

The analysis of the survival curves (Figure 7C) confirmed the efficacy of dinutuximab beta with an ILS of 68% (*p* = 0.0006 vs. vehicle). In the doxorubicin group, a comparable ILS of 85.7% was recorded (*p* = 0.0023 vs. control). The superimposable activity of the two active compound is confirmed also by the absence of significant difference between their survival curves (*p* = 0.725). The two combined treatments further improved mice survival, resulting in an ILS of 146% (*p* = 0.000025 vs. vehicle) and 150% (*p* = 0.0122 vs. vehicle) for the simultaneous and sequential combination, respectively. However, the simultaneous combination was significantly better than either dinutuximab beta or doxorubicin as monotherapy, while the sequential combination was not. Complete statistical analysis of the tumor growth curves is reported in Table S5.

**Figure 8:**
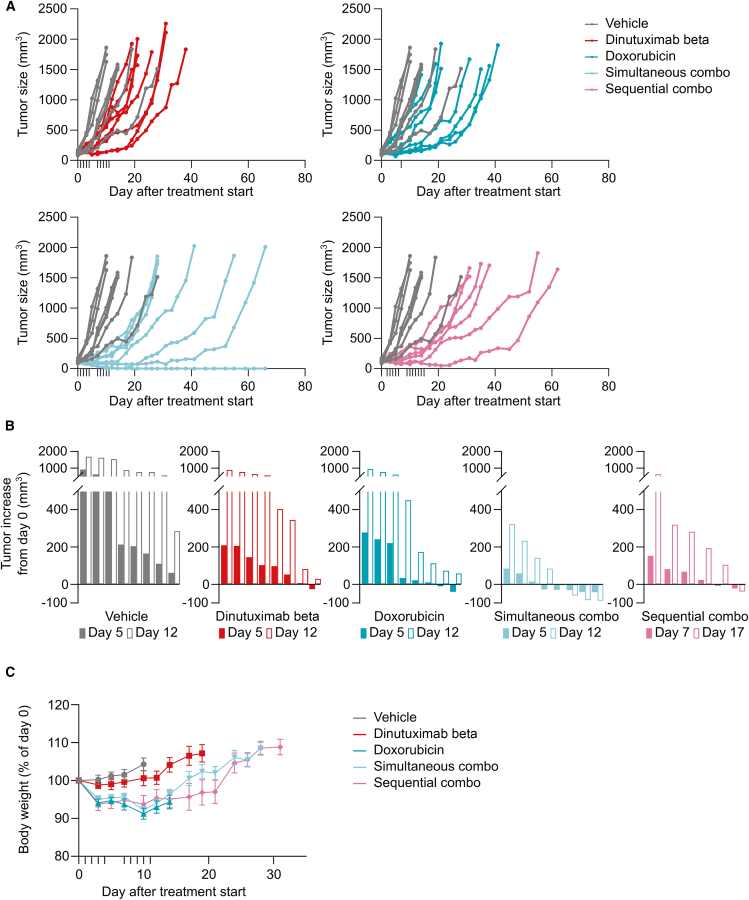
Two cycles of combination therapy induce tumor shrinkage (A) Growth curves of individual tumors. Tumor size is expressed as mm^3^. (B) Change in tumor size for each tumor at day 5 (solid bars) and day 12 (open bars) compared to day 0. For the sequential combination group, tumor size at day 7 (solid bars) and day 17 (open bars) is shown. Each bar represents the size of one tumor. Data are presented as the difference between tumor size on the indicated day (in mm^3^) and tumor size on day 0 (in mm^3^). (C) Plot showing average body weight of TC-71 tumor bearing mice treated with dinutuximab beta, doxorubicin and their combination. Data are shown in grams.

Figure 8 shows the growth curve of each individual tumor over time. All the tumors in the control group increased in size over the treatment period. On day 5, after the end of the first cycle of treatment, Dinutuximab beta and doxorubicin monotherapies caused partial shrinkage of one (12.5%) and two (25%) out of eight tumors, respectively. The “simultaneous combination” induced regression of five out eight tumors (62.5%) on day 5, which lasted for four out of eight (50%) tumors on day 12, at the end of the second cycle of treatment (Figure 8B). Conversely, the “sequential combination” was not as effective, inducing partial shrinkage in only one out six (16.7%) tumors. Subsequently, tumors in the two combination groups re-grew slower than those in the monotherapies groups, with one tumor in the simultaneous combination group being completely cured at the end of the observation period. Two cycles of treatment with dinutuximab beta were well tolerated, with no BWL or other drug-related clinical signs of distress (Figure 8C). As previously reported, doxorubicin caused significant BWL, averaging around 10%, both as monotherapy and in combination. In the “sequential combination” group, one mouse was euthanized due to tumor ulceration, and three other mice reported a BWL greater than 10%, resulting in a total of four out of seven animals (57%) experiencing health issues. Conversely, only two out eight mice (25%) in the “simultaneous combination” group reported a BWL greater than 10%. Taken together, these data suggest a better tolerability of the simultaneous combination regimen.

## Discussion

In this study, we investigated the effects of dinutuximab beta, a monoclonal antibody against the GD2 ganglioside, in combination with doxorubicin, a widely used chemotherapeutic agent, on EwS xenografts in mice. We found that dinutuximab beta is as effective as doxorubicin in reducing tumor growth and increasing survival of the animals. Moreover, we found that the combination of chemo and immunotherapy further increased the therapeutic efficacy of the single therapies leading to a stronger inhibition of tumor growth.

Administered in a single cycle of five consecutive daily injections, dinutuximab beta induced rapid tumor regression starting from the first day after therapy initiation. This rapid therapeutic response could imply an initial activation of complement, which typically acts more quickly than effector cells like NK cells. However, once the treatment course concluded, the tumor began to re-grow. A second cycle of treatment with dinutuximab beta resulted in only a slight improvement in its effectiveness, showing almost no inhibition of tumor growth. On the other hand, when combined with chemotherapy, the second cycle significantly enhanced therapeutic efficacy, resulting in slower tumor progression and prolonged survival. Collectively, these findings indicate that combining dinutuximab beta with doxorubicin overcomes the loss of therapeutic efficacy of dinutuximab beta when administered in multiple cycles, suggesting a synergistic effect that enhances antitumor efficacy. Histopathological analysis of tumors revealed that, while all treatments induced diffuse necrosis, doxorubicin, either alone or in combination, it also induced apoptosis. This may provide a rationale for the synergistic efficacy observed in chemo-immunotherapy.

The RNAseq analysis revealed the infiltration of effector cells of the innate immune response, likely driven by pro-inflammatory cytokines, in tumors treated with dinutuximab beta. These findings confirm the proposed mechanism of action of dinutuximab beta, which involves the recruitment of effector cells, mainly NK cells, to tumor sites. Here, effector cells exert their antitumor effect by inducing tumor cell death. The murine model used in the present study has some limitations. Although nude athymic mice retain a certain level of effector cells, including NK cells, the overall functionality of the innate immune response may be compromised compared to that of immunocompetent organisms. Consequently, the therapeutic efficacy of dinutuximab beta observed in this model may underestimate its potential in patients with fully functional immune systems.

In this study, we examined the effectiveness of two different chemo-immunotherapy regimens by giving doxorubicin and dinutuximab beta either at the same time or one after the other. The data indicate that administering them simultaneously results in stronger tumor reduction with slightly reduced toxicity.

In clinical practice, following clinical trial outcomes and SIOPEN guidelines, dinutuximab beta is administered to high-risk neuroblastoma patients as monotherapy after an intense consolidation phase of chemotherapy. Accordingly, Spasov and colleagues administered dinutuximab beta after each cycle of chemotherapy in patients with EwS.^9^ Our findings indicate that administering dinutuximab beta concurrently with doxorubicin enhances tumor response. If confirmed by clinical studies, this evidence can change the future approach of chemo-immunotherapy treatment for this tumor.

Overall, this study showed that dinutuximab beta is effective against EwS, especially in combination with doxorubicin, a standard component of the induction chemotherapy regimen for EwS. Further studies are needed to confirm the safety and efficacy of this combination in clinical trials.

In conclusion, the promising results of our preclinical experiments suggest a potential new therapeutic strategy for patients with EwS. By combining immunotherapy with traditional chemotherapy, it is possible to enhance treatment outcomes and improve patient survival rates. We eagerly anticipate the translation of these findings into clinical practice, paving the way for more effective cancer treatments.

### Limitations of the study

The main limitation of this study is the use of immunocompromized nude athymic mice. Although they retain certain effector cell populations, their innate immune functionality is likely impaired compared to immunocompetent organisms. This immunological impairment may have led to an underestimation of the therapeutic potential of dinutuximab beta, which could be substantially greater in patients with functional immune systems.

## Resource availability

### Lead contact

Further information and requests for resources and reagents should be directed to and will be fulfilled by the lead contact, Matteo Malinverno (malinverno.ma@recordati.it).

### Materials availability

This study did not generate new unique reagents.

### Data and code availability

• The RNA-seq data generated in this study have been deposited in GEO and are available for download under accession number GSE310619 at https://www.ncbi.nlm.nih.gov/geo/query/acc.cgi?acc=GSE310619
• This article does not report original code.
• Any additional information is available from the lead contact upon request.

## Acknowledgments

We thank Dr. Valter Torri for statistical analysis support and Dr. Fabio Frigerio for his excellent support for antibodies conjugation. This study was sponsored by Recordati S.p.A.

## Author contributions

M. Malinverno and S.B. conceived the project and designed the experiments together with R.F.; R.F., M. Meroni, E.B., L.P., E.C., V.K., and I.P. performed the experiments and collected and analyzed the data; S.C. and E.S. conducted histopathological evaluation; M. Malinverno wrote the manuscript, which has been revised by all of the authors.

## Declaration of interests

L.P., E.C., V.K., I.P., P.A., S.B. and M. Malinverno are employees of Recordati S.p.A. M. Malinverno and S.B. have a patent application related to this work. The other authors declare no conflict of interest.

## STAR★Methods

### Key resources table

**Table:** 

REAGENT or RESOURCE	SOURCE	IDENTIFIER
**Antibodies**		

Dinutuximab beta	Recordati S.p.a.	N/A
human IgG1 isotype control	BioXcell	cat. BE0297; RRID:AB_2687817
PE Mouse Anti-Human Disialoganglioside GD2	BD Biosciences	Cat. 562100; RRID:AB_11154036
PE Mouse IgG2a, κ Isotype Control	BD Biosciences	Cat. 559319; RRID:AB_397217

**Chemicals, peptides, and recombinant proteins**		

Doxorubicin	MedChemExpress	cat. HY-15142

**Critical commercial assays**		

CellTiter-Glo Luminescent Viability Assay KIT	Promega	Cat. G7571
BD PE QuantiBrite Beads	BD Biosciences	Cat. 340495
CellMask Deep Red Plasma Membrane Stain	ThermoFisher	Cat. C10046
ADCC Reporter Bioassay, Core Kit	Promega	Cat. G7018
IgG Human SimpleStep ELISA® Kit	AbCam	Cat. ab195215
Trizol Reagent	Invitrogen	Cat. 15596026
PureLink RNA Mini Kit	Life technologies	Cat. 12183018A
Oxford Nanopore Technologies PCR-cDNA Sequencing Kit	ONT	Cat. SQK-PCB114-24

**Deposited data**		

Raw RNA-seq data	This paper	https://www.ncbi.nlm.nih.gov/geo/query/acc.cgi?acc=GSE310619

**Experimental models: Cell lines**		

TC-71 human Ewing sarcoma cell line	Laboratory Cancer Pharmacology of Istituto di Ricerche Farmacologiche Mario Negri	N/A
CHO-K1 cells	ATCC	Cat. CCL-61
SK-N-BE(2) human neuroblastoma cell line	ATCC	Cat. CRL-2271

**Experimental models: Organisms/strains**		

Female Athymic nude mice, 7–8 weeks	INOTIV	Cat. 6904F

**Software and algorithms**		

GraphPad Prism 10	GraphPad Software, Inc.	https://www.graphpad.com/
SAS® 9.4	SAS Institute	https://support.sas.com/downloads
MinKNOW acquisition software (v24.11.10).	Oxford Nanopore Technologies	Software downloads | Oxford Nanopore Technologies
fastpLong v0.3.0	N/A	https://doi.org/10.1002/imt2.107
minimap2 v2.30	N/A	https://doi.org/10.1093/bioinformatics/bty191
Xenomapper	N/A	https://doi.org/10.21105/joss.00018
Salmon v1.10.3	N/A	https://doi.org/10.1038/nmeth.4197
ComBat_seq function/sva package v3.52.0	N/A	https://doi.org/10.1093/nargab/lqaa078
DESeq2 v1.44.0	N/A	https://doi.org/10.1186/s13059-014-0550-8
RNAenrich: A web server for non-coding RNA enrichment	Innovative Drug Research and Bioinformatics Group (IDRB)	https://idrblab.cn/rnaenrich/

### Experimental model and study participant details

#### Cell line

TC-71 cell line carrying the EWS/FLI1 type I translocation were available at the Laboratory Cancer Pharmacology of Istituto di Ricerche Farmacologiche Mario Negri. The cells were maintained in Iscove’s modified Dulbecco’s medium supplemented with 10% Fetal Bovine serum, 2 mM glutamine, and 1% Penicillin-Streptomycin. CHO-K1 cells were purchased from ATCC and maintained in a 1:1 mixture Minimum Essential Medium and Ham’s F12 Medium supplemented with 10%Fetal Bovine Serum (FBS) and 1% Penicillin-Streptomycin. SK-N-BE(2) neuroblastoma cells were purchased form ATCC and cultured in a 1:1 mixture of Eagle’s Minimum Essential Medium and F12 Medium supplemented with 10% Fetal Bovine serum and 1% Penicillin-Streptomycin. All the cell lines were tested for mycoplasma contamination upon arrival at the laboratory and maintained in line for the minimum number of passages required for execution of the experiments.

#### Mice

All the procedures with animals were performed in agreement with the Institutional Animal Care and Use Committee (IACUC) of Istituto di Ricerche Farmacologiche Mario Negri IRCCS (IRFMN), in compliance with the guidelines established in the Principles of Laboratory Animal Care (Directive 86/609/EEC) and as approved by the Italian Ministry of Health (Aut. Min n. 646/2022-PR).

Female Athymic nude mice, 7–8 weeks old, were obtained from INOTIV (Netherlands). They were maintained under Specific Pathogen Free conditions with constant temperature and humidity and fed with a regular chow diet and water *ad libitum*. Female mice were used for experimental practicality, as they can be randomised without exhibiting aggressive behavior. Sex has no influence on the study results, as Ewing sarcoma is not sex-related and the antibody’s mechanism of action is sex-independent relying solely on GD2 expression.

### Method details

#### Drug treatment and cell viability assay

Cell viability was assessed using the CellTiter-Glo Luminescent Viability Assay KIT (Promega, Madison, WI, USA). TC-71 cells, at 80% confluence, were washed with PBS, trypsinized and then seeded at the density of 10,000 cells per well in a 96-well white plate previously coated with Matrigel (Corning) diluted 1:100 in Dulbecco’s medium (Gibco). Cells were cultured in complete medium (Iscove’s modified Dulbecco’s medium with 10% Fetal bovine serum and 1% Penycillin/Streptomycin) at 37°C with 5% CO2. After 24 h, the cells were treated with serial dilution of Dinutuximab beta (starting from 4.3 μM), doxorubicin (0.2% DMSO; starting from 300 μM) in basal medium. After 24 and 48 h of incubation, an equal volume of CellTiter-Glo reagent to the volume of cell culture medium present in each well was added, and the plate was incubated for 10 min to stabilize the luminescent signal. The luminescence was then measured using the FlexStation 3 Multi-Mode Microplate Reader (Molecular Devices, San Jose, CA, USA). Relative cell viability was calculated as follows: cell viability at the given concentration/cell viability of control × 100. The experiment was conducted three times, each with three technical replicates.

#### Fluorescent-activated cell sorting (FACS)

10^6^ TC-71 cells were resuspended in 100ul of PBS with 2% FBS + 2 mM EDTA and then incubated for 30 min at 4°C with Dinutuximab beta conjugated with the fluorophore IRDye800CW. After three washes the cells were fixed with 1% PFA and then acquired on a Cytek Aurora cell sorter. Negative controls were TC-71 cells unstained.

#### ABC (antibodies bound per cell) analysis

SK-N-BE(2) and TC 71cells were grown to 90% confluency and detached using trypsin. 1x10^6^cells/ml per tube were washed in cold FACS buffer (PBS supplemented with 2% heat-inactivated FBS and 2 mM EDTA) followed by incubation with anti-GD2-PE antibody (final concentration: 3 μg/mL; Clone 14G2a, Isotype: mouse IgG2a, BD Biosciences) or isotype control mouse IgG2a-PE,k (Isotype: control mouse IgG2a-PE,k; BD Biosciences) for 45 min at 4 °C, protected from light. Cells were then washed twice with FACS buffer and transferred to filter top tubes with addition of DAPI live/dead stain (final concentration: 1 μg/mL; Sigma-Aldrich). Data were acquired using a Cytek Aurora flow cytometer, collecting 20,000 events from the live (DAPI-negative) singlet gate for each cell line. BD PE QuantiBrite Beads (BD Biosciences) were analyzed according to the manufacturer’s protocol during each run. A standard curve was generated in GraphPad Prism, from which the PE geometric mean for each sample was converted into the number of antibodies bound per cell (ABC), representing surface antigen expression. Mean and S.E.M. were calculated from a minimum of three replicate values for each cell line.

#### Immunofluorescence and confocal imaging

TC-71 cells were fixed with 4% Paraformaldehyde and then incubated with Rhodamine Red-X conjugated Dinutuximab beta for 2 h at room temperature, without permeabilization. Cells were stained with CellMask Deep Red Plasma Membrane Stain (ThermoFisher) for 10 min, washed with PBS, fixed in 4% paraformaldehyde for 10 min at room temperature, and mounted with DAPI-containing medium for nuclei counterstain. Images were acquired with a IX81 confocal (Olympus, Tokyo, Japan).

#### ADCC

Antibody-dependent cellular cytotoxicity was evaluated with the ADCC Reporter Bioassay, Core Kit, form Promega (Cat. G7018), following manufacturer’s instructions. A ratio effector to target cells equal to 10:1 was used. As negative controls, both IgG isotype on TC-71 cells and Dinutuximab beta on CHO-K1 (GD2 negative) cells were used.

#### Tumor models

To obtain the tumors, 200 μL of cell suspension containing 5 x 10^6^ TC-71 cells were injected subcutaneously into the right flanks of athymic nude mice. The growing tumor masses were measured with the aid of a Vernier caliper, and the tumor volume (mm^3^) was calculated by the formula: length x (width)^2^/2.

#### Antitumor activity study in xenograft models

When tumor volume reached about 100 mm^3^, mice were randomized into the treatment groups. Tumor bearing mice were monitored at least twice a week by Vernier caliper. Mice were euthanized when tumors reach a volume of ≥1500 mm^3^, in case of tumor ulceration or body weight loss >20%. The antitumor activity was expressed as T/C%, where T and C are the mean tumor volume of treated and control groups, respectively.

Parallel groups of 4 mice for each treatment group were sacrificed *ad interim* at the indicated time points. Terminal blood samples (approx. 400 μL) were collected, and plasma-separated by centrifugation at 4000 rpm at 4°C for 10 min. Tumors were removed and immediately frozen in dry-ice or formalin fixed paraffine embedded for pharmacodynamic analysis.

#### Pharmacokinetic study and IgG dosage

Blood was collected in EDTA-coated tubes, kept at 4°C and centrifuged for 10 min at 4000 RPM. Plasma was immediately frozen at −20°C until the analysis. Plasma concentration of Dinutuximab beta was determined with an ELISA kit for human IgG from AbCam (IgG Human SimpleStep ELISA Kit, Cat. ab195215), according to manufacturer’s instructions.

#### Histology

Subcutaneous tumors were harvested and immediately fixed in 10% formalin for 24h, then put in 70% ethanol until paraffin embedding, following standard procedure. 5 μm thick sections were obtained at the microtome and stained with haematoxylin and eosin (H&E).

Histopathological evaluation: samples were examined with a light microscope for the detection of histological features of the tumor. Histopathology evaluation was made in a blind fashion, i.e., without knowledge of the treatment group. Four animals for each satellite group were analyzed. For necrosis, the percentage of affected tissue was given; moreover, each specimen was assigned a score depending on the primary distribution pattern of necrosis. The score system was arbitrary defined as follows.

**Table:** 

Pattern of Necrosis Distribution	Assigned Value
Central	0
Diffuse throughout the tumor, predominantly central	1
Diffuse throughout the tumor	2
Diffuse throughout the tumor, predominantly peripheral	3
Peripheral	4

The count of apoptotic and mitotic figures was made in three randomly selected microscopy fields, avoiding the areas of necrosis.

#### RNA extraction

Frozen tumor sections collected at T1 and T2, were homogenized using TissueLyser LT (Qiagen, Benelux, B.V.) in Trizol Reagent (Invitrogen, Cat. No.15596026). The PureLink RNA Mini Kit (Life technologies Cat. No. 12183018A) was adopted to extract and purify total RNA. The concentration and purity of RNA were determined at 260/280 nm using Nano Drop One Spectrophotometer (Thermo Fisher Scientific Inc. USA).

#### RNA-seq Library preparation and sequencing

Bulk long-read RNA sequencing was performed using the Oxford Nanopore Technologies (ONT; Oxford, UK) PCR-cDNA Sequencing Kit (SQK-PCB114-24). Sequencing libraries were prepared according to the manufacturer’s protocol. Briefly, 700 ng of full-length RNA per sample was used as input for complementary DNA (cDNA) synthesis and strand switching with kit-supplied oligonucleotides. Double-stranded cDNA (dscDNA) was generated by PCR amplification (10 μL cDNA/sample) using primers containing 5′ tags, which enable ligase-free attachment of rapid sequencing adapters. The resulting libraries were loaded onto R10.4.1 flow cells (FLO-PRO114M) and sequenced on P2 Solo PromethION devices using MinKNOW acquisition software (v24.11.10).

#### RNA-seq raw data processing

Raw ONT sequencing data in POD5 format were basecalled using Dorado v7.6.8 with the high-accuracy (HAC) model v4.3.0 (400 bps) within MinKNOW (v24.11.10). Basecalled reads were subjected to quality control and reads filtering with fastpLong v0.3.0.^19^ Reads in which >40% of bases had a Phred score <15 were removed to ensure downstream data quality. High-quality reads were aligned to both the Gencode human transcriptome (GRCh38, v38) and the Gencode mouse transcriptome (GRCm39, vM28) using minimap2 v2.30^20^ with the *map-ont* preset optimized for ONT long reads. Xenomapper^21^ was used to segregate human- and mouse-derived reads in xenograft samples. Transcript-level quantification was then performed using Salmon v1.10.3^22^ with parameters --gcBias --numBootstraps 10 -L U to account for GC bias and provide bootstrap estimates of variance. Transcript-level abundance estimates were aggregated to gene-level raw counts with tximport v1.32.0.^23^ Batch effects were corrected using the ComBat_seq function from the sva package v3.52.0.^24^ Finally, genes with low counts for a number of samples equal to the minimum group size were filtered out (<10 counts for human, <5 counts for mouse, reflecting differences in read depth).

#### Exploratory analysis

For exploratory analyses, raw counts were normalized with DESeq2 v1.44.0^25^ and variance-stabilized using the rlog() function. Principal Component Analysis (PCA) of normalized expression values was performed with prcomp() from the stats package v4.4.1. Only results with an adjusted *p*-value <0.05 were retained for visualization.

#### Differential expression and functional enrichment

Differentially expressed genes (DEGs) were identified using DESeq2 v1.44.0, with significance defined as an adjusted *p*-value <0.05 after multiple testing correction (Benjamini–Hochberg).

Functional enrichment analysis of DEG was performed using RNAenrich.^26^ The Gene Ontology (GO) term Biological Process was interrogated.

### Quantification and statistical analysis

#### Statistical analysis

Tumor growth curves were analyzed using GraphPad Prism version 9 software (GraphPad software, Inc., La Jolla, CA, USA) performing generalized linear mixed-effects models. This test gives the same results as repeated measures ANOVA if there are no missing values, and comparable results when there are missing values.

For the survival curves statistical analysis was performed with SAS 9.4 software. The median and range were calculated for the survival time. A Restricted Mean Survival Time (RMST) analysis (Royston and Parmar BMC Medical Research Methodology 2013, 13:152) comparing the area under the Kaplan-Meier survival curves (AUC), calculated up to the last experimental observation, was performed. For the data from the histopathological evaluation the Kruskal–Wallis’s test followed by the Dunn’s post hoc test was applied.
