# Supplementary material for: Dinutuximab beta effectively treats Ewing sarcoma when combined with chemotherapy
Source: iScience. 2025 Dec 16;29(1):114449. doi: 10.1016/j.isci.2025.114449 (PMC12803847; doi:10.1016/j.isci.2025.114449)
Supplement: Document S1. Figures S1–S5 and Tables S1–S5 [file mmc1.pdf]

iScience, Volume *29*

Supplemental information

Dinutuximab beta effectively treats Ewing sarcoma when combined with chemotherapy

Roberta Frapolli, Marina Meroni, Ezia Bello, Lorenza Pirona, Elisa Callegari, Valentina Kebede, Isabella Pellerani, Simone Canesi, Eugenio Scanziani, Uta Dirksen, Patrizia Angelico, Stefano Biondi, and Matteo Malinverno

Table S1. Statical analysis of tumour growth curves, related to Figure 2b.

Mixed-effects model (REML)	p-value
Vehicle vs Control IgG	0.241
Vehicle vs Dinutuximab Beta	**0.0343**
Vehicle vs Doxorubicin	**0.0215**
Vehicle vs Combination	**0.0027**
Dinutuximab beta vs Doxorubicin	0.3511
Dinutuximab beta vs Combination	**0.0067**
Doxorubicin vs Combination	0.2394

Table S2. Statistical analysis of survival curves, related to Figure 2c.

RMST (vs Vehicle)	DIFF.	ST.ERR.	Z	p-value
**Control IgG**	-1.67	2.67	-0.62	0.5327
**Dinutuximab beta**	10.67	3.76	2.84	**0.0046**
**Doxorubicin**	19.67	6.08	3.23	**0.0012**
**Combination**	19.78	3.57	5.55	**0.0000**
**Dinutuximab beta vs Doxorubicin**	9.00	6.78	1.33	0.1843

RMST (vs Combination)	DIFF.	ST.ERR.	Z	p-value
**Dinutuximab beta**	9.11	4.66	1.95	0.0506
**Doxorubicin**	0.11	6.67	0.02	0.9868

Table S3. Histopathological evaluation of necrosis, related to Figure 4.

Timepoint and group	ID	Necrosis %	Necrosis distribution; assigned value
T1 Vehicle	835	10%	Central; 0
	859	10%	Central;0
	872	10%	Mostly central; 1
	878	10%	Mostly central; 1
	**mean**	**10%**	**0.5**
T1 Ctrl IgG	806	80%	Mostly peripheral;3
	833	30%	Diffuse; 2
	849	30%	Diffuse; 2
	871	30%	Diffuse; 2
	**Mean**	**40%**	**2.25**
T1 Dinutuximab	803	20	Central; 0
	831	20%	Diffuse; 2
	839	50%	Mostly central; 1
	869	80%	Diffuse; 2
	**Mean**	**42,5%**	**1.25**
T1 Doxorubicin	817	40%	Mostly peripheral; 3
	821	60%	Diffuse; 2
	868	40%	Diffuse; 2
	886	40%	Mosly central; 1
	**Mean**	**45%**	**2**
T1 Combination	808	40%	Mostly central; 1
	829	20%	Mostly central; 1
	844	50%	Diffuse; 2
	879	40%	Diffuse; 2
	**Mean**	**37.5%**	**1.5**
T2 Vehicle	855	30%	Mostly central; 1
	857	60%	Mostly central; 1
	864	50%	Mostly central; 1
	875	40%	Mostly central; 1
	**mean**	**45%**	**1**
T2 Ctrl IgG	807	60%	Mostly peripheral; 3
	811	40%	Mostly peripheral; 3
	814	40%	Diffuse; 2
	884	70%	Mostly central; 1
	**Mean**	**52.5%**	**2.25**
T2 Dinutuximab	830	70%	Diffuse; 2
	858	70%	Diffuse; 2
	866	70%	Diffuse; 2

	889	80%	Diffuse; 2
	**Mean**	**72.5%**	**2**
T2 Doxorubicin	809	80%	Mostly peripheral; 3
	825	40%	Diffuse; 2
	842	40%	Peripheral; 4
	843	40%	Mostly peripheral; 3
	**Mean**	**50%**	**3**
T2 Combination	816	80%	Diffuse; 2
	840	80%	Diffuse; 2
	852	70%	Diffuse; 2
	860	90%	Diffuse; 2
	**Mean**	**80**	**2**

Figure legend

\ = no pathological finding(s)

Severity: + = slight; ++ = moderate; +++ = severe

**Pattern of Necrosis Distribution**	**Assigned Value**
Central	0
Diffuse throughout the tumor, predominantly central	1
Diffuse throughout the tumor	2
Diffuse throughout the tumor, predominantly peripheral	3
Peripheral	4

Representative H&E images are displayed in **Figure S2.**

Table S4. Statical analysis of tumour growth curves of the two-cycle treatment, related to Figure 7b.

Mixed-effects model (REML)	p-value
Vehicle vs Dinutuximab beta	**0.0135**
Vehicle vs Doxorubicin	**0.0189**
Vehicle vs Combination simultaneous	**0.0009**
Vehicle vs Combination sequential	**0.0018**
Dinutuximab beta vs Doxorubicin	0.7417
Dinutuximab beta vs combination simultaneous	**0.0106**
Dinutuximab beta vs combination sequential	**0.0248**
Doxorubicin vs combination simultaneous	**0.0481**
Doxorubicin vs combination sequential	0.102
Combination simultaneous vs combination sequential	0.832

Table S5. Statistical analysis of survival curves of the two-cycle treatment, related to Figure 7c.

RMST (vs Vehicle)	DIFF.	ST.ERR.	Z	p-value
**Dinutuximab beta**	11.13	3.22	-3.45	**0.0006**
**Doxorubicin**	12.63	4.15	3.04	**0.0023**
**Combination simultaneous**	27.63	6.56	4.21	**0.000025**
**Combination sequential**	19.38	7.73	2.51	**0.0122**
**Doxorubicin vs Dinutuximab beta**	1.50	4.26	0.35	0.7250

RMST (vs Dinutuximab beta)	DIFF.	ST.ERR.	Z	p-value
**Combination simultaneous**	16.5	6.64	2.49	**0.0129**
**Combination sequential**	8.25	8.00	1.03	0.3026

RMST (vs Doxorubicin)	DIFF.	ST.ERR.	Z	p-value
**Combination simultaneous**	15	7.13	2.10	**0.0354**
**Combination sequential**	6.75	8.22	0.82	0.4115

Figure S1

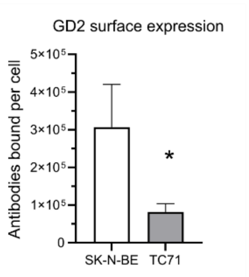

Figure S1. GD2 expression in TC-71 cells, related to Figure 1.

GD2 surface expression (ABC = antibodies bound per cell) as measured by flow cytometry and quantified with BD Quantibrite beads. Data are presented as mean ± S.E.M. , $p< 0.05$ (Student's t-test)

Figure S2

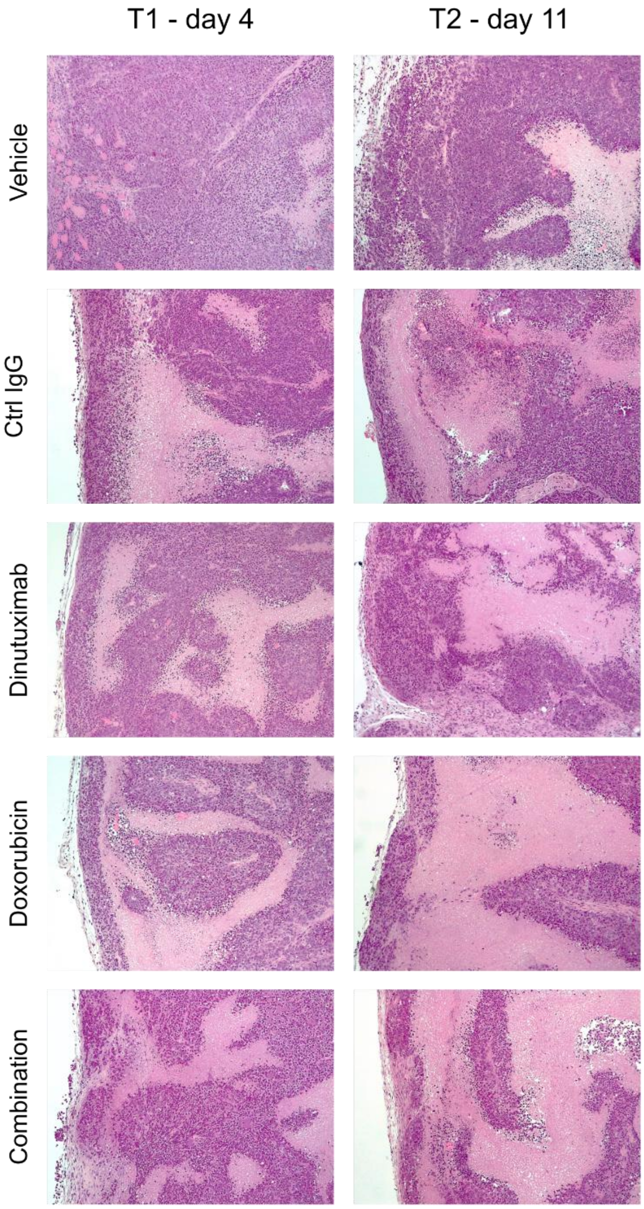

Figure S2. Spatial distribution of necrosis.

Representative H&E images showing the distribution of necrosis across tumor specimen under different conditions; scale bar = 50µm. The histological evaluation is reported in **Table S3.**

Figure S3

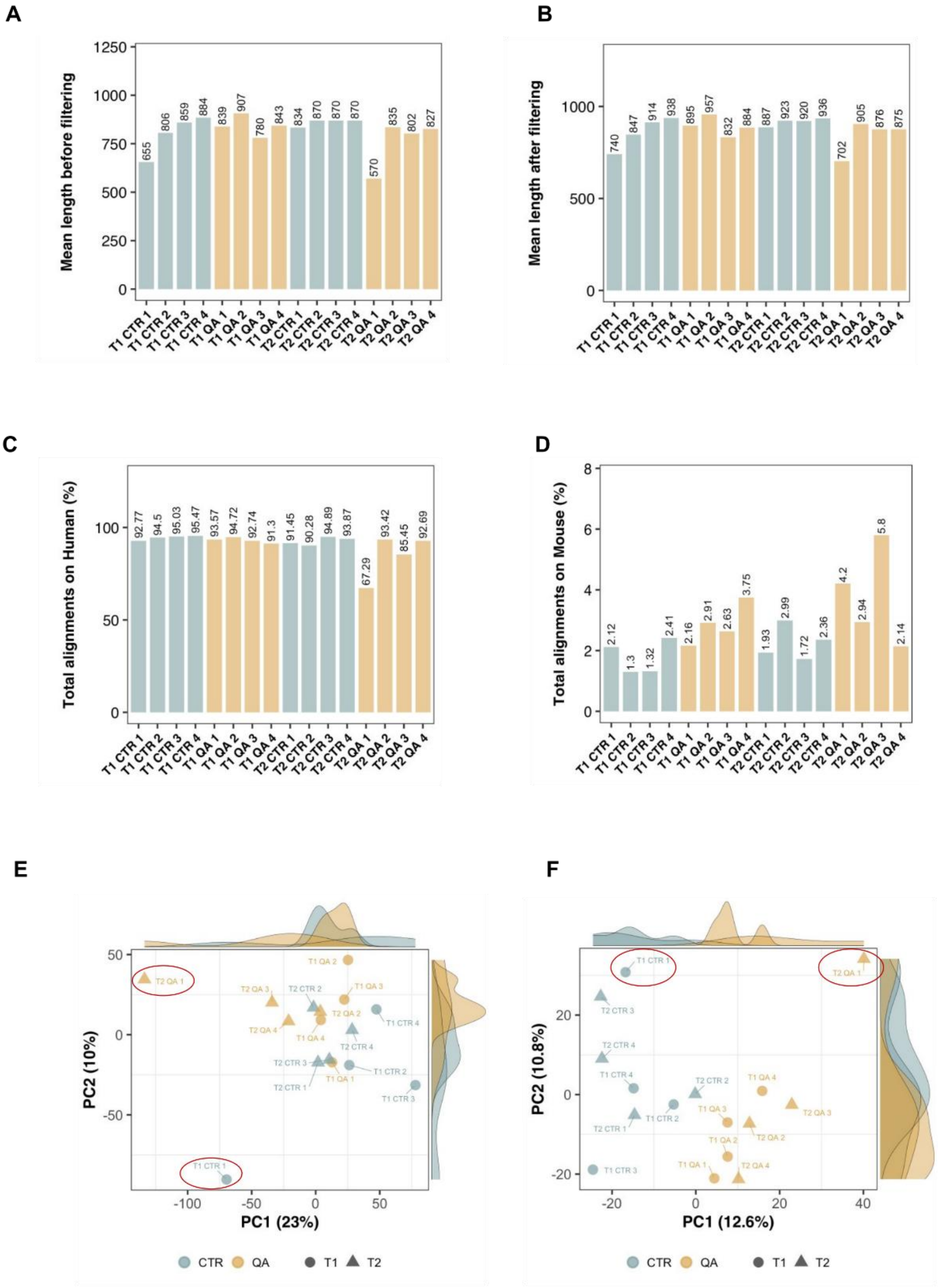
A
Mean length before filtering
655
806
859
884
839
907
780
843
834
870
870
870
570
835
802
827
T1 CTR 1
T1 CTR 2
T1 CTR 3
T1 CTR 4
T1 QA 1
T1 QA 2
T1 QA 3
T1 QA 4
T2 CTR 1
T2 CTR 2
T2 CTR 3
T2 CTR 4
T2 QA 1
T2 QA 2
T2 QA 3
T2 QA 4
B
Mean length after filtering
740
847
914
938
895
957
832
884
887
923
920
936
702
905
876
875
C
Total alignments on Human (%)
92.77
94.5
95.03
95.47
93.57
94.72
92.74
91.3
91.45
90.28
94.89
93.87
67.29
93.42
85.45
92.69
D
Total alignments on Mouse (%)
2.12
1.3
1.32
2.41
2.16
2.91
2.63
3.75
1.93
2.99
1.72
2.36
4.2
2.94
5.8
2.14
E
PC1 (23%)
PC2 (10%)
F
PC1 (12.6%)
PC2 (10.8%)
CTR
QA
T1
T2

Figure S3. Sequencing metrics and Principal component analysis

A-B) Bar plot showing reads length for each sample before (**A**) and after **(B**) filtering. T1 = time point 1; T2 = time point 2; CTR = vehicle; QA = Dinutuximab beta. Samples T1_CTR_1 and T2_QA_1 display reduced reads length, likely due to partial degradation. **C.D**) Bar plot showing the percentage of alignments on human (**C**) and murine (**D**) genome for each sample. **E-F)** Principal component analysis showing the distribution of each sample for the human (**E**) and murine (**F**) genome.
Samples T1_CTR_1 and T2_QA_1 exhibited strong separation from their respective experimental groups in the PCA space, indicating potential technical anomalies. Based on their anomalies both in the reads length and PCA analysis, these samples were therefore excluded from downstream differential expression analyses to ensure robust comparisons between groups.

Figure S4

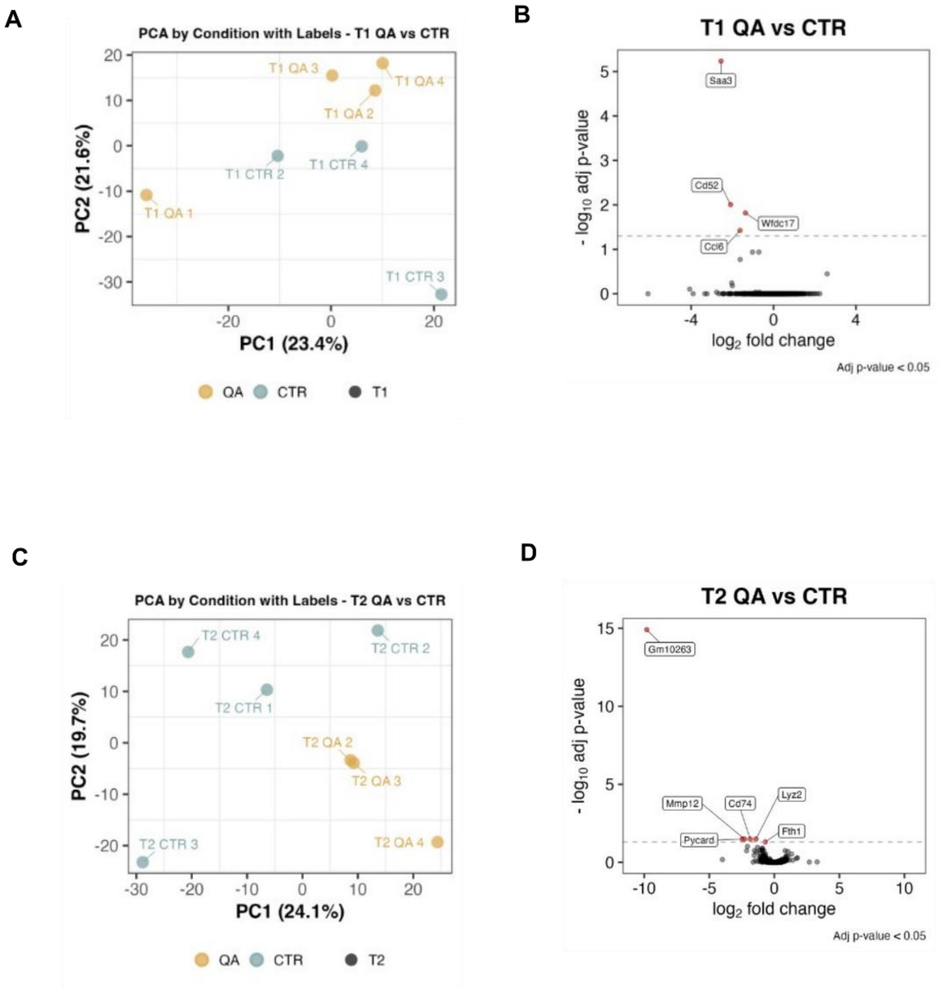

Figure S4. Analysis on murine genome.

A, C) Principal component analysis at time points1 (**A**) and 2 (**C**). CTR = vehicle; QA = Dinutuximab beta. **B, D)** Volcano plots showing differentially expressed transcripts from Dinutuximab beta and vehicle treated tumors at T1 (**B**) and T2 (**D**). CTR = vehicle; QA = Dinutuximab beta. Only results with an adjusted p-value <0.05 are visualized.

Figure S5

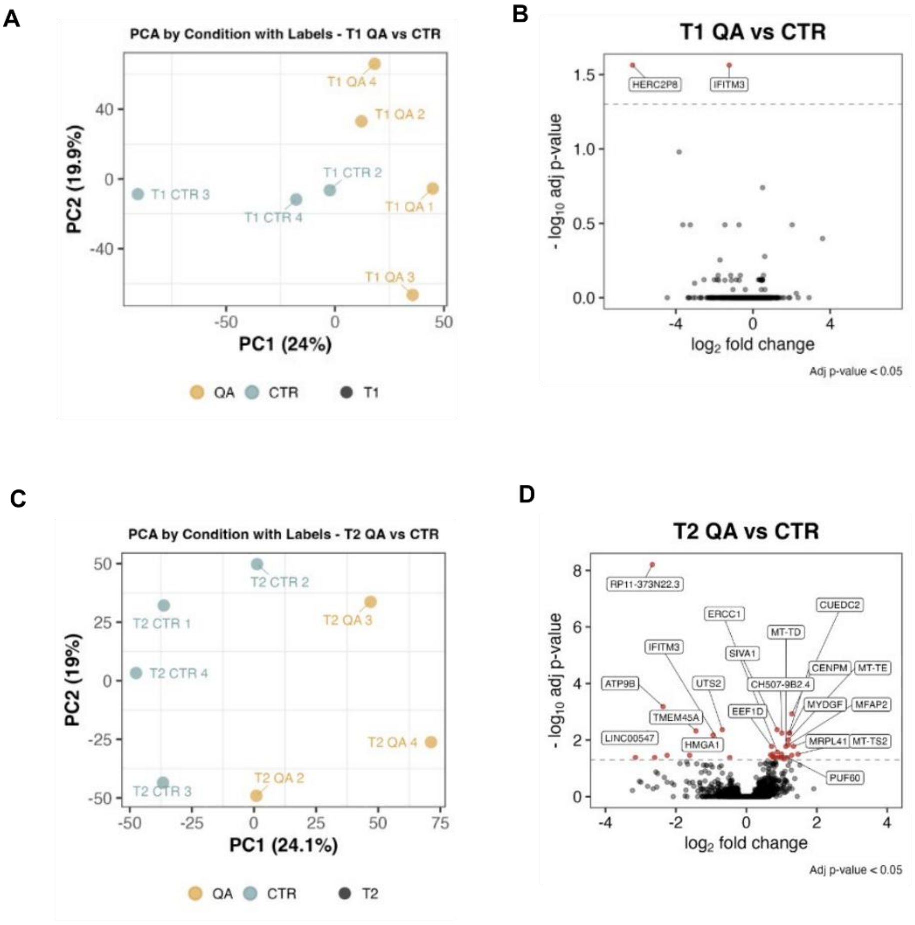

Figure S5. Analysis on human genome.

A, C) Principal component analysis at time points1 (**A**) and 2 (**C**). CTR = vehicle; QA = Dinutuximab beta. **B, D)** Volcano plots showing differentially expressed transcripts from Dinutuximab beta and vehicle treated tumors at T1 (**B**) and T2 (**D**). CTR = vehicle; QA = Dinutuximab beta. Only results with an adjusted p-value <0.05 are visualized.
